# Identification of critical prognosis signature associated with lymph node metastasis of stomach adenocarcinomas

**DOI:** 10.1186/s12957-023-02940-y

**Published:** 2023-02-23

**Authors:** Xiaohui Wang, Wei Zhang, Yulin Guo, Yifei Zhang, Xiaofeng Bai, Yibin Xie

**Affiliations:** 1 Department of General Surgery, Xuanwu Hospital, Capital Medical University, Beijing, 100053 China; 2 Department of Pancreatic and Gastric Surgery, National Cancer Center/National Clinical Research Center for Cancer/Cancer Hospital, Chinese Academy of Medical Sciences and Peking Union Medical College, 100021 Beijing, China

**Keywords:** Data mining, Prognosis, Biological maker, Expression difference, Functional enrichment analysis

## Abstract

Lymph node metastasis (LNM) is an important factor affecting the prognosis of patients with gastric adenocarcinoma (STAD), which is the most common malignancy of the human digestive system. Current detection techniques have limited sensitivity and specificity, and there is a lack of effective biomarkers to screen for LNM. Therefore, it is critical to screen for biomarkers that predict LNM in STAD. Gene expression differential analysis (false discovery rate < 0.05, |log_2_Fold change| ≥1.5) was performed on 102 LNM samples, 224 non-LNM samples, and 29 normal gastric tissue samples from The Cancer Genome Atlas (TCGA) STAD dataset, and 269 LNM-specific genes (DEGs) were obtained. Enrichment analysis showed that LNM-specific genes functioned mainly in cytokine-cytokine receptor interactions, calcium signaling, and other pathways. Ten DEGs significantly associated with overall survival in STAD patients were screened by multivariate Cox regression, and an LNM-based 10-mRNA prognostic signature was established (Logrank *P* < 0.0001). This 10-mRNA signature was well predicted in both the TCGA training set and the Gene Expression Omnibus validation dataset (GSE84437) and was associated with survival in patients with LNM or advanced-stage STAD. Using Kaplan-Meier survival, receiver operating characteristic curve, C-index analysis, and decision curve analysis, the 10-mRNA signature was found to be a more effective predictor of prognosis in STAD patients than the other two reported models (*P* < 0.0005). Protein-protein interaction network and gene set enrichment analysis of the 10-mRNA signature revealed that the signature may affect the expression of multiple biological pathways and related genes. Finally, the expression levels of prognostic genes in STAD tissues and cell lines were verified using *q*RT-PCR, Western blot, and the Human Protein Atlas database. Taken together, the prognostic signature constructed in this study may become an indicator for clinical prognostic assessment of LNM-STAD and provide a new strategy for future targeted therapy.

**Supplementary Information:**

The online version contains supplementary material available at 10.1186/s12957-023-02940-y.

## Background

Gastric cancer (GC) is the fifth most common malignancy in the world and is an important factor affecting patient prognosis [1]. Of these, gastric adenocarcinoma (STAD), which accounts for 95% of cases, is the most common histological type of gastrointestinal malignancy. Lymph node metastasis (LNM) in STAD is the result of a combination of multiple biological pathways driving the disease progression [2–4]. Currently, the limited sensitivity and specificity of the LNM screening method and the lack of validated biomarkers largely limit the effect of diagnosis and treatment of STAD [5–7]. Therefore, there is an urgent need to screen for specific markers that can predict STAD LNM to further guide treatment.

Second-generation sequencing technology is used to rapidly identify tumor characteristics, based on which appropriate cancer treatment strategies can be designed [8]. With the development and application of sequencing technology in the past decades, the large-scale high-throughput data have become an effective resource for finding cancer biomarkers. Zhang et al. used bioinformatics analysis to construct a profile of five miRNAs in GC and found that the target genes of these miRNAs are involved in various cancer-related pathways [9]. A novel genome-wide 11-miRNA signature predicting recurrence in GC patients was identified [10]. An expression signature consisting of 10 angiogenesis-related genes (ITGAV, STC1, APOH, SLCO2A1, NRP1, POSTN, VTN, SERPINA5, LPL, KCNJ8) was shown to predict the prognosis of GC patients [11]. Researchers constructed a prognostic model including four genes (RASSF2, MS4A2, ANKRD33B, and ADH1B) to predict patient LNM based on The Cancer Genome Atlas (TCGA) STAD dataset [12]. However, the study was conducted only for the immune microenvironment and was not validated using an external dataset. Molecular markers or prognostic models associated with LNM have been reported in other cancer studies. A combination of four miRNAs (miR-502, miR-145, miR-142, and miR-33b) was found to serve as independent prognostic features in cervical cancer (CRC). Among them, miR-502 and miR-33b together with two LNM key genes (PTPRC and CDH5) could be used as potential novel biomarkers for CRC [13]. Eight immune-related gene signatures (IRGs), including IKBKB, LTBR, MIF, PPARD, PPIA, PSME3 S100A6, and SEMA4B, were associated with LNM in lung adenocarcinoma (LUAD) and were constructed as a reliable risk scoring model [14]. Luo et al. screened APOL2, AHNAK, GSDMB, and SHTN1 by co-expression analysis and constructed a prognostic model for bladder cancer (BC) [15]. Thirteen miRNAs as a disease signature can serve as a non-invasive method to objectively predict LNM in patients with LUAD [16].

**Fig. 1:**
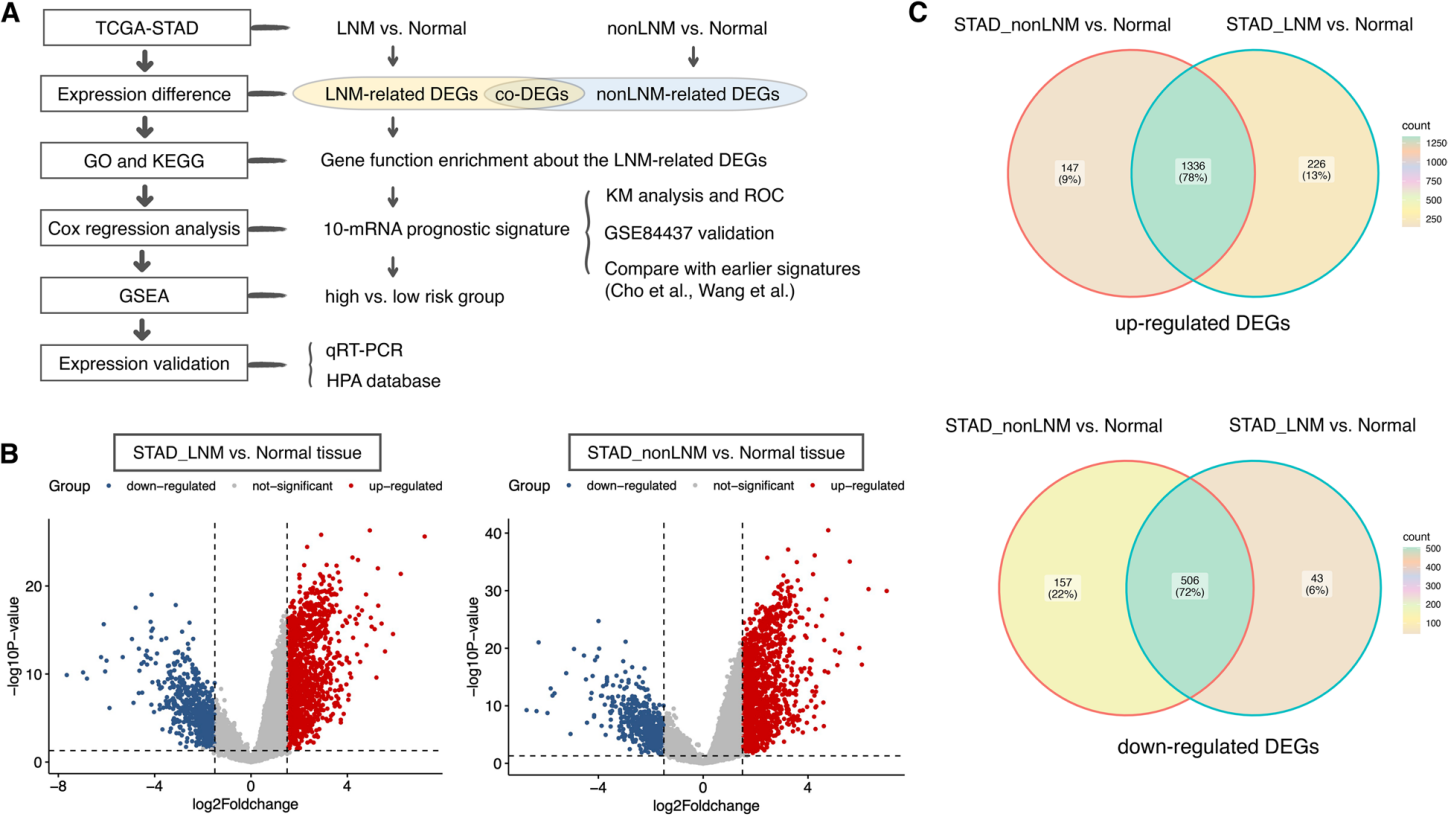
Analysis workflow and lymph node metastases related genes in STAD. **A** Experimental design and analytical workflow of this study. **B** Volcano plots of DEGs for LNM STAD samples versus normal gastric tissue (left) and non-LNM STAD samples versus normal gastric tissue (right), respectively. *x*-axis: log fold change; *y*-axis: -log 10 (*P* value) for each gene; vertical dotted line: fold change ≥1.5 or ≤−1.5; horizontal dotted line: significance cutoff point (*P* value = 0.05). Red dots represent upregulated genes and blue dots represent downregulated genes. **C** Venn diagram of the overlap between DEGs

In this study, a series of R language packages were used to perform differential gene expression analysis on the TCGA STAD dataset to obtain LNM-specific differentially expressed genes (DEGs). The main biological functions and the involved signaling pathways were explored by enrichment analysis of LNM-DEGs by Gene Ontology (GO) and Kyoto Encyclopedia of Genes and Genomes (KEGG). The prognostic signature of STAD based on LNM-DEGs was established by the Cox risk regression method, validated by Gene Expression Omnibus (GEO) external STAD dataset GSE84437, and compared with the reported prognostic features of GC for predicting the effect. Risk scores based on prognostic signatures were used to classify STAD patients into high and low-risk groups. The correlation between prognostic signature and clinical characteristics of STAD patients was investigated. Gene set enrichment analysis (GSEA) was also performed on differential genes between the high and low groups to explore the functional pathways associated with the 10-mRNA signature. The protein interaction (PPI) network was used to explore the interactions between the 10-mRNA signature. Finally, the expression of the prognostic signature was verified using *q*RT-PCR and Western blot in GC cell lines, tissue samples, and the Human Protein Atlas (HPA) database (Fig. 1A).

## Materials and methods

### Data collection and gene expression processing

High-throughput sequencing gene expression data and clinical information of STAD patients, including 326 STAD tissues and 29 normal gastric tissues, were obtained from the TCGA database download. According to the American Joint Commission (AJCC) clinicopathological grading criteria for STAD lymph node metastases (LNM) (N0~N3), the 326 patients were classified into the non-LNM group (N0, 102 cases) and the LNM group (N1~N3, 224 cases), respectively. Differential gene expression analysis was performed using the R language limma package for the non-LNM group vs. normal group and the LNM group vs. normal group, respectively [17]. The false discovery rate (FDR) < 0.05 and the absolute value of expression difference fold change (|log_2_FC|) ≥ 1.5 were used as thresholds for significant differences, and the analysis results were visualized with the ggplot2 and pheatmap packages [18]. LNM-specific DEGs, non-LNM-specific DEGs, and non-LNM and LNM co-expression DEGs (co-DEGs) for STAD were obtained using the R language VennDiagram package [19]. The TCGA-STAD dataset was also used as a training set to construct a risk regression signature of STAD patients based on LNM-specific DEGs. The STAD dataset GSE84437 sequencing information in the NCBI GEO database is based on GPL6947 (Illumina HumanHT-12 V3.0 Expression BeadChip) microarray platform, which contains 433 STAD samples (Table S1). The GSE84437 dataset was used as a test set to validate the validity and accuracy of the prognostic signature.

### Functional enrichment analysis

Based on Gene Ontology (GO) and Kyoto Encyclopedia of Genes and Genomes (KEGG) [20], the LNM-DEGs, non-LNM-specific DEGs, and co-DEGs obtained in the previous step were analyzed for biologically functional enrichment, including cell composition (CC), molecular function (MF), and biological processes (BP), as well as biological pathways, diseases, and drugs, using the ClusterProfile and ggplot2 packages in R language.

### Construction and evaluation of prognostic signature

Based on the clinical data of STAD patients in the TCGA dataset, univariate Cox proportional hazards regression analysis was performed using the Survival and Survminer packages in R to screen for statistically significant DEGs in STAD LNM-DEGs (*P*<0.05). Then, multivariate COX regression analysis was conducted using a bivariate stepwise regression method to screen out genes associated with the prognosis of STAD patients, and risk scores (RS) were calculated according to the following formula.

$$Risk\ score=\sum\limits_{i=1}^n{Coef}_i\ast {x}_i$$

Using the median RS value as the threshold, STAD patients were included in the high-risk and low-risk groups, respectively. The Kaplan-Meier method with Log-rank test was used to assess the survival of STAD patients. The validity and sensitivity of the prognostic characteristics were assessed by calculating the area under the receiver operating characteristic (ROC) curve (AUC) for STAD patients in the training and test groups. In addition, as the patients were divided into different clinical subgroups according to LNM, clinicopathology, histological grading, and age, the relationship between the prognostic characteristics and clinical factors in STAD patients was analyzed in the training and test datasets. Further, to assess the superiority of the prognostic characteristics of LNM-related STAD, the predictive performance of this model was compared with two other models reported in previous studies (5 gene signatures proposed by Wang et al. and 6 gene signatures proposed by Cho et al.) using K-M survival analysis, ROC curve analysis, Harrell consistency index (C-index), and decision curve analysis (DCA) [21, 22].

### Gene set enrichment analysis

To elucidate the molecular mechanisms of STAD involved in the LNM-related prognostic feature, DEGs were analyzed between high-risk and low-risk groups using the limma package in R. Based on KEGG, GO, and The Reactome Pathway database (https://reactome.org/), significantly enriched biological pathways and functions were analyzed by the GSEA method (*P* < 0.05). The analysis results were visualized using the clusterProfile package in R.

### Protein-protein interaction network construction

The protein-protein interaction (PPI) network of the genes in the constructed signature was mapped using The Search Tool for the Retrieval of Interacting Genes (STRING) website (http://string-db.org/) and visualized with Cytoscape software. The core module clusters were screened with the Cytoscape plugin, Molecular Complex Detection (MCODE), with the cutoff parameters of node score (0.2), degree (2), K-core (2), and max depth (100), and *P* value (0.05). The biological function of each module was annotated with the Local network cluster in STRING (*P* < 0.05).

### Patients and samples

To detect the signature expression in tumor tissues, we collected 20 pairs of GC tissues and matched adjacent normal tissues from patients who underwent radical surgery at the Cancer Hospital of the Chinese Academy of Medical Sciences (CAMS) between June 2020 and April 2022. The patients were diagnosed with lymph node metastases, and none of them received neoadjuvant chemoradiotherapy. All patients signed an informed consent form for the use of samples. The Human Ethics and Research Ethics Committees of the Cancer Hospital, the CAMS approved the study (approval no. 14-067/857).

### Cell culture

Gastric cancer cell lines (AGS and MGC-803) were purchased from the Shanghai Institute of Cell Biology, Chinese Academy of Sciences. The cell lines were cultured in Dulbecco’s modified Eagle medium (DMEM; Invitrogen, Carlsbad, CA, USA) containing 10% fetal bovine serum (FBS; HyClone, Logan, UT, USA), 100 U/ml penicillin and 100 mg/ml streptomycin in a 37°C, 5% CO_2_ incubator.

### Expression verification of LNM-related prognostic signature

The total RNA was extracted using a Trizol reagent (Invitrogen, CN). cDNA was then synthesized using the Advantage RT-for-PCR Kit (Clontech), diluted, and subjected to *q*RT-PCR using the HiScript® II One Step *q*RT-PCR SYBR ® Green Kit (Takara, Japan). *q*RT-PCR was performed (Table S2). *GADPH* was used as an internal control for mRNA, and the relative expression levels of genes were calculated by the 2^-ΔΔCt^ method.

Protein was extracted with RIPA lysis buffer containing protease inhibitors and measured with a standard bovine serum albumin (BSA) kit. The extracted proteins were separated electrophoretically using 10% sodium dodecyl sulfate-polyacrylamide gels (SDS-PAGE) and then transferred to polyvinylidene difluoride (PVDF) membranes (Millipore Corporation, Billerica, MA, USA). The membranes were blocked with 5% non-fat milk for one hour at room temperature and then incubated with the primary antibody (PA5-114294, Invitrogen, California, USA; GTX121032, GeneTex, Texas, USA; ab137118 ab80264, ab276749, ab42108, ab288419, ab249907, ab283897, and ab8245, Abcam, Cambridge, UK) at 4°C overnight and then washed with TBST solution (Boster, China). These membranes were then incubated with secondary antibodies (bs-0295G, BIOSS, China). Finally, ECL chemiluminescence detection system is used for signal detection. Protein expression profiles in normal gastric tissues or STAD tissues were also obtained using the Human Protein Atlas (HPA, https://www.proteinatlas.org/) online tool.

## Results

### Identification of DEGs related to lymph node metastasis of STAD

Differential gene expression analysis was performed for LNM tissues (*n*=102) and non-LNM tissues (*n*=224) versus normal tissues (*n*=29), respectively. The results showed that 1562 genes were significantly upregulated and 549 genes were downregulated in LNM STAD tissues compared with normal tissues, while 1483 genes were significantly upregulated and 663 genes were significantly downregulated in non-LNM STAD tissues (Fig. 1B). Overlapping analysis of DEGs showed that 226 upregulated genes and 43 downregulated genes were specifically expressed in LNM tissues (Table S3), 146 upregulated genes and 157 downregulated genes were specifically expressed in non-LNM tissues, while 1336 upregulated genes and 506 downregulated genes were co-differentially expressed in LNM and non-LNM tissues (Fig. 1C).

### Functional enrichment analysis

**Fig. 2:**
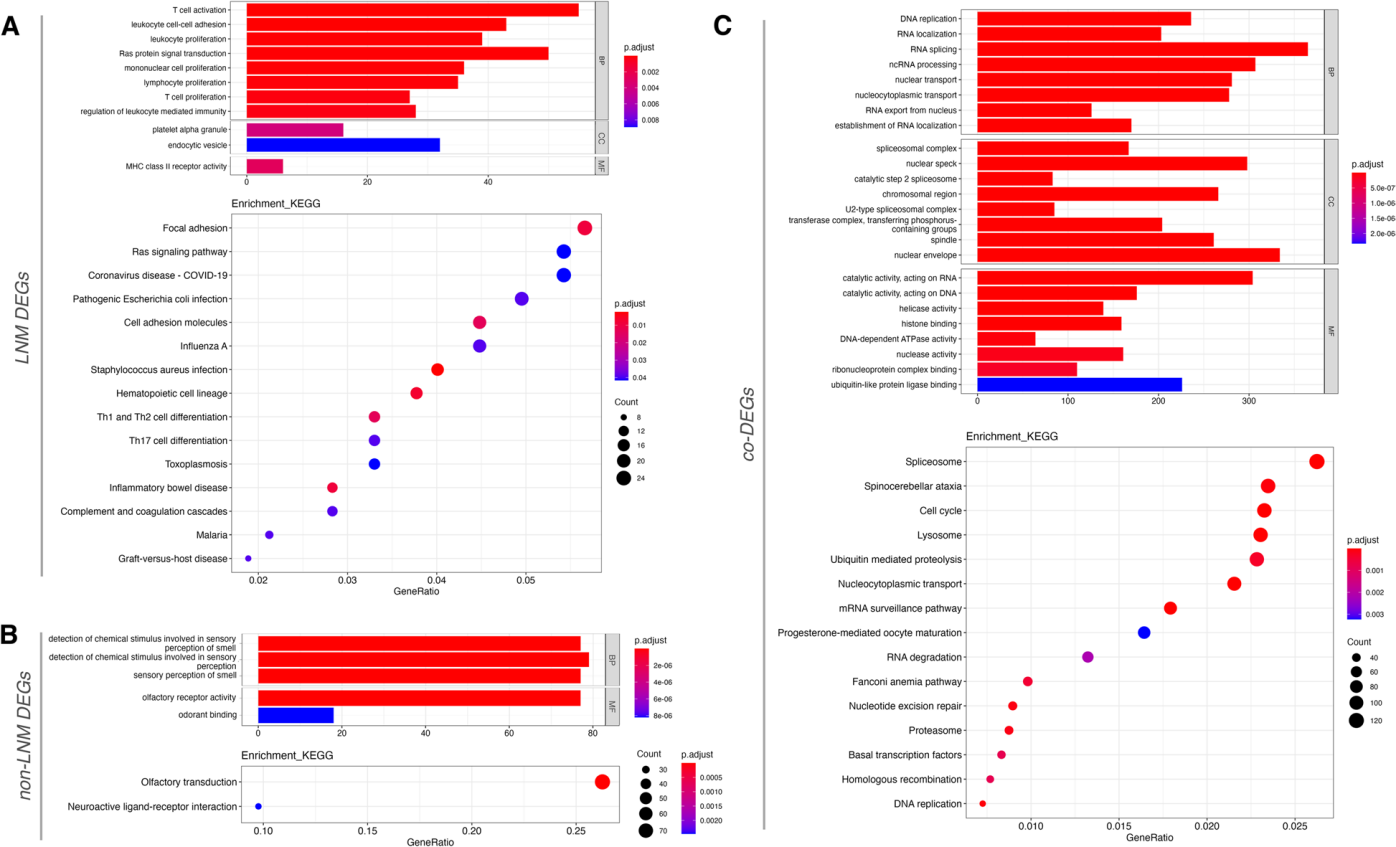
Functional enrichment analysis of GO and KEGG for differentially expressed genes. Based on the results of the previous step, the LNM-DEGs (**A**), nonLNM-DEGs (**B**), and co-DEGs (C) were functionally annotated for GO categories including biological processes (BP), molecular functions (MF), and cellular components (CC) and KEGG biological pathways, respectively

GO functional annotation of LNM-specific DEGs, non-LNM-specific DEGs, and co-DEGs, respectively, revealed that LNM-specific DEGs were mainly enriched in leukocyte cell adhesion (GO:0007159, *P*<0.0001), platelet *α* granules (GO 0031091, *P*=0.003), MHC class II receptor activity (GO 0032395, *P*=0.002), etc. (Fig. 2A; Table S4); non-LNM-specific DEGs were mainly enriched in chemical stimulus detection (GO 0050911, *P*=2.44E−30), olfactory receptor activity (GO 0004984, *P*=1.46E−29), etc. (Fig. 2B; Table S5); co-DEGs were mainly enriched in DNA replication (GO 0006260, *P*=5.75E−25), spliceosome complex (GO 0005681, *P*=1.21E−23), catalytic activity, acting on RNA (GO 0140098, *P*=6.04E−15), etc. (Fig. 2C; Table S6).

KEGG pathway enrichment analysis showed that LNM-specific DEGs were mainly enriched in hematopoietic cell lines (hsa04640, *P*=0.007), *Staphylococcus aureus* infection (hsa05150, *P*=0.002), and inflammatory bowel disease (hsa05321, *P*=0.009) (Fig. 2A; Table S7); non-LNM-specific DEGs were mainly enriched in olfactory transduction (hsa04080, *P*=2.74E−31), neuroactive ligand-receptor interactions (hsa04080, *P*=0.002) (Fig. 2B; Table S8); co-DEGs were mainly enriched in nucleocytoplasmic transport (hsa03013, *P*=1.03E−13), cell cycle (hsa04110, *P*=3.84E−10), and spliceosomes (hsa03040, *P*=1.46E−09) (Fig. 2C; Table S9).

### Establishment of multigene prognostic signature

To investigate the effect of LNM-DEGs on patient survival, we did univariate Cox regression analysis on 269 LNM-DEGs and obtained 24 genes with *P* values less than 0.05 (Fig. S1), and then conducted multivariate Cox regression analysis and subsequently obtained 10 genes with *P* values less than 0.05 (Fig. S1). The results showed that these 10 genes were associated with the OS of STAD patients, including RAI14 (HR=0.376, *P*<0.001), GUCY1A2 (HR=0.524, *P*=0.001), CNGB3 (HR=0.572, *P*=0.003), FJX1 (HR=0.662, *P*=0.026), FZD2 (HR=0. 591, *P*=0.006), and ELOVL2 (HR=0.641, *P*=0.017) were probably benign prognosis, while CXCL13 (HR=1.606, *P*=0.012), GDPD4 (HR=1.602, *P*=0.011), TIGIT (HR=1.636, *P*=0.010), and SEL1L3 (HR=1.542, *P*=0.017) may have a poor prognosis.

**Fig. 3:**
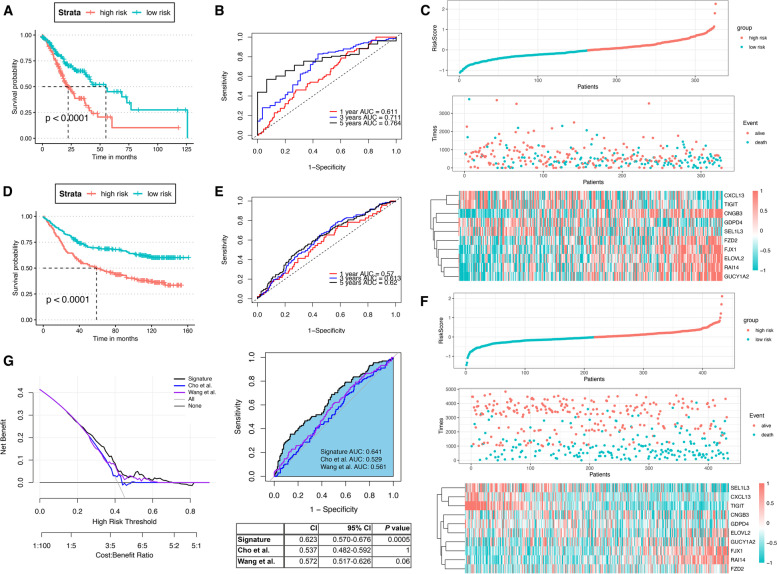
A prognostic feature containing ten mRNAs constructed based on LNM-DEGs. **A** KM survival analysis of the high-risk and low-risk groups in the TCGA-STAD dataset. **B** Time-dependent ROC curves of patients’ OS at 1, 3, and 5 years in the TCGA-STAD dataset. **C** Survival status, survival time (days), and relationship between risk score classes in high- and low-risk groups, and expression patterns of ten genes in the TCGA-STAD dataset. **D** KM survival analysis between the high- and low-risk groups in the GSE83347 dataset. **E** Time-dependent ROC curves at the 1, 3, and 5 years for patients’ OS of the GSE83347 dataset. **F** Survival status, survival time (days), and relationship between risk score classes in high- and low-risk groups, and expression patterns of ten genes in the GSE83347 dataset. **G** ROC curves and DAC profiles in the TCGA-STAD dataset comparing the performance of 10-mRNA signatures compared to previous signatures, including Cho’s and Wang’s gene signatures

Therefore, a 10-mRNA risk-prognosis model was constructed based on these 10 genes. The median RS value (1.049) calculated according to the prognostic scoring formula (RS = −0.308 × RAI14 expression −0.166 × SEL1L3 expression + 0.142 × GUCY1A2 expression + 0.119 × CXCL13 expression - 0.004 × CNGB3 expression + 0.034 × GDPD4 expression + 0.029 × FJX1 expression + 0.079 × FZD2 expression + 0.008 × TIGIT expression + 0.042 × ELOVL2 expression), dividing the 326 patients into low- and high-risk groups. As shown in Fig. 3A, the survival rate in the low-risk group was significantly higher than that in the high-risk group (Logrank *P* < 0.0001). Subsequently, the survival rates of STAD patients at 1, 3, and 5 years were assessed using the 10-mRNA signature, and the time-dependent ROC curves showed AUC values of 0.611, 0.711, and 0.764, respectively, which implied that the prognostic signature had the good prognostic ability (Fig. 3B). Figure 3C showed the gene expression of the prognostic signature in the high-risk and low-risk groups. As the prognostic score increased, the number of deaths in STAD patients increased, indicating that the higher the risk score, the worse the prognosis of STAD patients.

### Validation and comparison of a prognostic signature

Subsequently, the accuracy of the 10-mRNA prognostic signature was validated in the GSE84437 dataset. The 433 STAD patients were divided into low-risk and high-risk groups based on the median RS value (0.948) calculated from the prognostic score formula. As shown in Fig. 3D, STAD patients in the low-risk group had significantly higher OS than those in the high-risk group (*P*<0.0001). The AUC values of 0.570, 0.613, and 0.620 for the 1-year, 3-year, and 5-year survival rates of patients analyzed according to 10 genetic prognostic traits indicated that the trait also had a good prognostic ability (Fig. 3E). The higher the prognostic score, the more STAD patients died, indicating that the higher the risk score, the worse the prognosis of STAD patients in the GSE84437 dataset (Fig. 3F). Several prognostic models have been identified in previous studies to predict the survival of STAD patients. In the present study, the predictive effect of 10 gene signatures with the predictive performance of two previous models was further done. For normalization, the gene expression levels in each model were extracted uniformly from the original matrix of the TCGA-STAD dataset. Based on the corresponding coefficients provided by each model, the risk score of each STAD patient was calculated accordingly, and the patients were divided into high-risk and low-risk groups. As shown in Fig. 3G, by comparing the ROC curves, CI values (95% CI values), and DCA curves, the results showed that the AUC of the 10-mRNA model constructed in this study was higher and more stable than the other features, and the C-index was also the highest among the three models. This further demonstrated the better clinical utility of the 10-mRNA signature in predicting the survival of STAD patients.

### Kaplan-Meier analysis in clinical subgroups

**Fig. 4:**
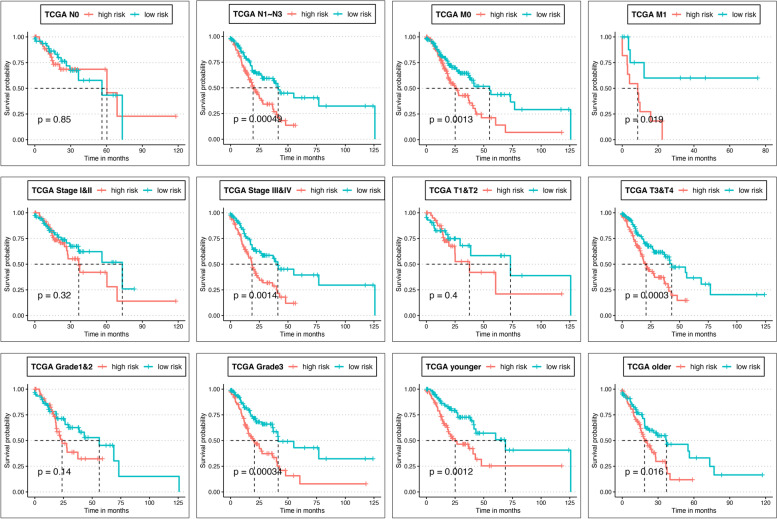
KM analysis of high- and low-risk groups in relation to the TCGA dataset (**A**) and the GSE84437 clinical subgroup (**B**), respectively

Next, we investigated the relationship between 10-mRNA characteristics and OS in TCGA-STAD patients in different clinical subgroups. As shown in Fig. 4, in the clinical subgroups of LNM stage (N1~N3), clinical stage (III~IV), tumor grading T3~T4, and advanced pathological grading (G3~G4) groups, the OS of STAD patients in the low-risk group was significantly higher than that in the high-risk group. In contrast, in the clinical subgroups of non-LNM (N0), early clinical stage (I~II), tumor grading T1~T2, and early pathological grading (G1~G2) groups, there was no significant difference in survival between STAD patients in the high-risk and low-risk groups. In both the distal metastasis and age clinical subgroups, the overall survival rate of STAD patients in the low-risk group was significantly higher than that in the high-risk group.

### Gene set enrichment analysis

**Fig. 5:**
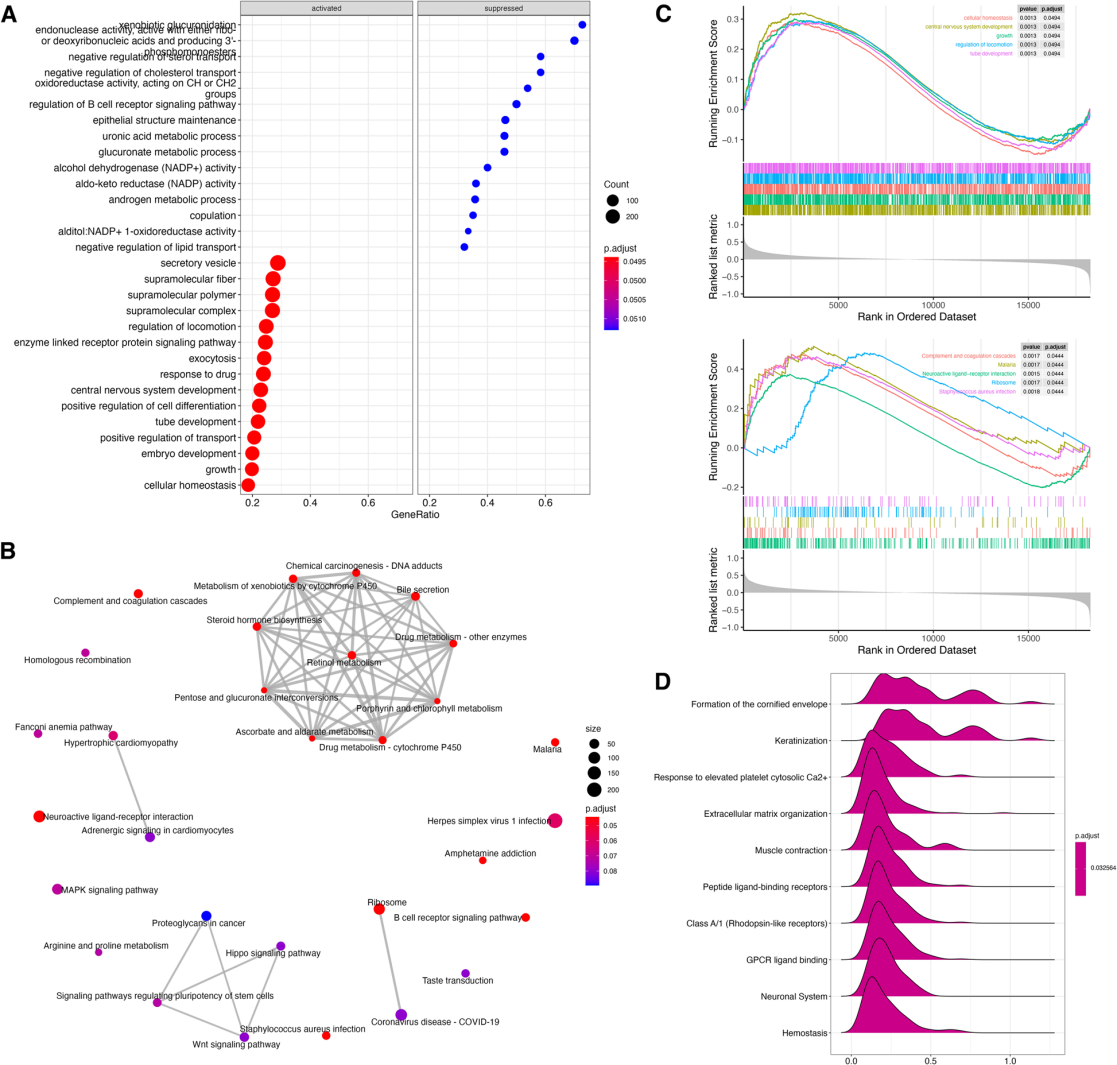
Functional enrichment and annotation analysis of DEGs between the high- and low-risk groups by the ten-mRNA signature. **A** The dotplot showing GO enrichment analysis. **B** The emaplot showing KEGG pathway enrichment analysis. **C** GSEA enrichment analysis. **D** The ridgeplot showing Rectome pathway enrichment analysis; *P* value less than 0.05 indicated a significant enrichment term

To explore the possible involvement of the 10-mRNA signature in tumor biological pathways, we performed differential gene expression analysis for the high- and low-risk groups and obtained 709 DEGs, including 320 genes with upregulated expression and 389 genes with downregulated expression. GSEA analysis of DEGs showed (Fig. 5A; Table S10) that the results indicated that the expression of secretory vesicles (GO 0099503, *P*=0.001), supramolecular fibers (GO 0097435, *P*=0.002), supramolecular polymers (GO 0099081, *P*=0.001), and other related genes were activated in STAD tissues. In contrast, the expression of genes associated with xenobiotic glucuronidation (GO 0052697, *P*=0.002), negative regulation of sterol transport (GO 0032372, *P*=0.002), and regulation of B cell receptor signaling pathway (GO 0050855, *P*=0.001) were suppressed in STAD tissues. KEGG enrichment analysis revealed that major KEGG pathways included retinol metabolism (hsa00830, *P*=0.002), chemotaxis-DNA adducts (hsa05204, *P*=0.001), and drug metabolism-other enzymes (hsa00983, *P*=0.002) (Fig. 5B; Table S11). Figure 5C shows the top 5 enriched 10-mRNA signatures that may be involved in tumor biological pathways. The major enriched Rectome pathways include the formation of a keratinized envelope (R-HSA-6809371, *P*=0.001), keratinization (R-HSA-6805567, *P*=0.001), response to elevated platelet cell membrane Ca^2+^ (R-HSA-76005, *P*=0.002), etc. (Fig. 5D; Table S12).

### Protein-protein interaction

**Fig. 6:**
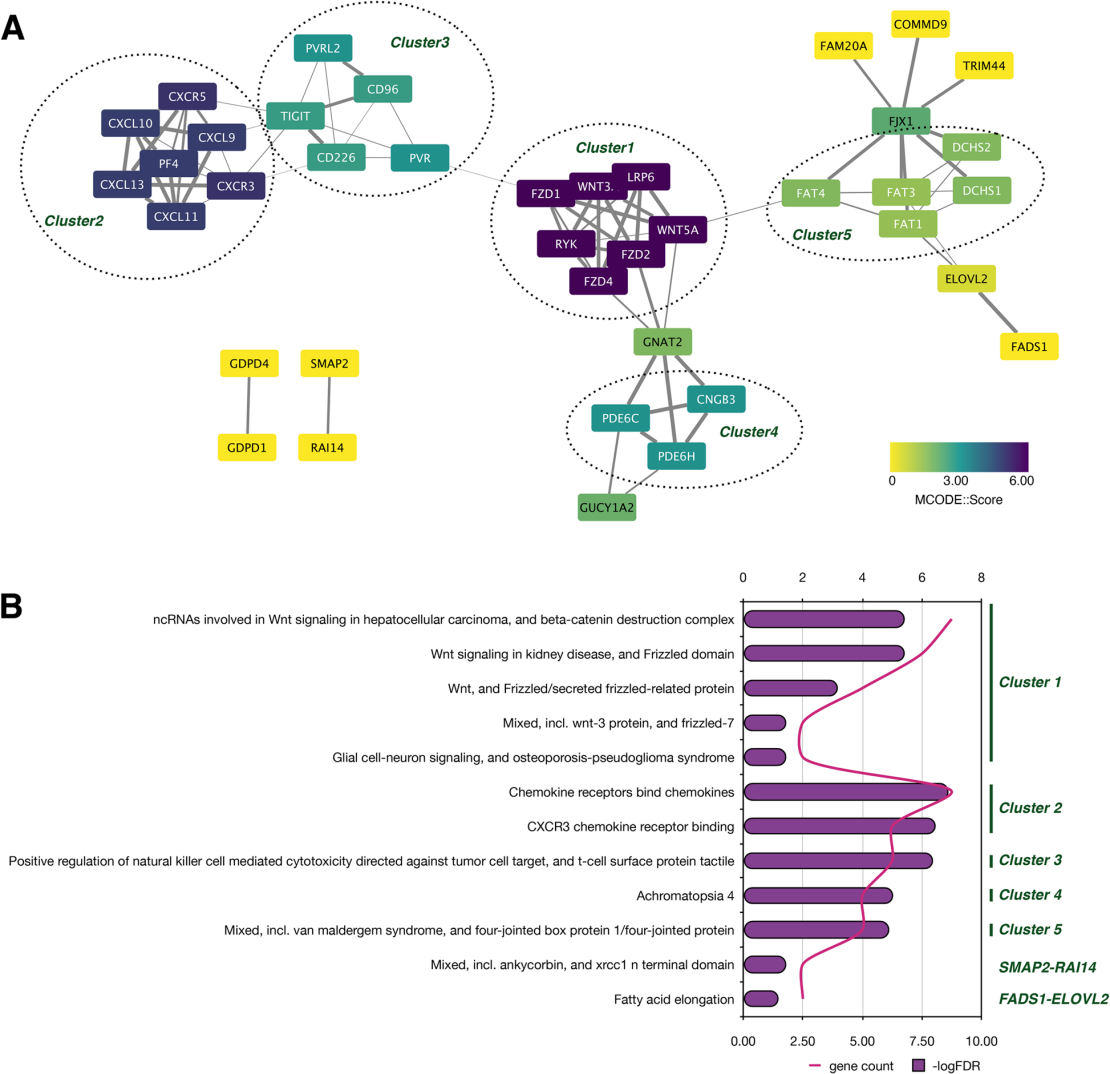
Protein-protein interaction (**A**) and functional enrichment analysis (**B**) of the ten-mRNA signature. **A** The dashed circles indicate the clusters analyzed by the MCODE module. Protein colors show the MCODE scores, yellow to purple indicates 0 to 6, and similar branches indicate the same cluster. Gray edges between proteins indicate that the proteins connected at both ends have reciprocal relationships, and the thickness indicates the combined score between 0 and 1, with the thicker indicating a higher score. **B** Histogram of STRING cluster functional enrichment analysis of the protein network

To investigate the functional relationships between ten-mRNA signatures, we performed a PPI analysis of ten-mRNA signatures and constructed a network of 40 proteins containing 9 proteins in ten-mRNA signatures except for SEL1L3 (Table S13). The protein network consisted of 86 edges, with each protein interacting with at least 4 proteins on average (*P*<1.0e−16). As shown in Fig. 6A, the cluster analysis was performed using the MCODE plugin in Cytoscape software, and a total of 5 Clusters were obtained. Functional enrichment analysis showed (Fig. 6B, Table S14) that Cluster 1 was mainly enriched in the Wnt signaling pathway (CL 21042, *P*=1.64E−07; CL 21046, *P*=1.64E−07; CL 21049, *P*=0.00011; CL 21052, *P*=0.02), cluster 2 was mainly enriched in chemokine receptors binding (CL 18276, *P*=2.48E−09; CL:18318, *P*=8.87E-09), cluster 3 was mainly enriched in positive regulation of natural killer cell mediated cytotoxicity directed against tumor cell target, and t cell surface protein tactile (CL 19158; *P*=1.13E−08), cluster 4 was mainly enriched in Achromatopsia 4 (CL 24112; *P*=5.27E−07), and the cluster 5 was mainly enriched in mixed, incl. van maldergem syndrome, and four-jointed box protein 1/four-jointed protein (CL 22077; *P*=7.8E−07). In addition, the proteins which were not classified in clusters, SMAP2-RAI14 were mainly associated with mixed, incl. ankycorbin, and xrcc1 N terminal domain (CL:25376; *P*=0.02), and GDPD4-GDPD1 were related to fatty acid elongation (CL 14207; *P*=0.02).

### Expression validation

**Fig. 7:**
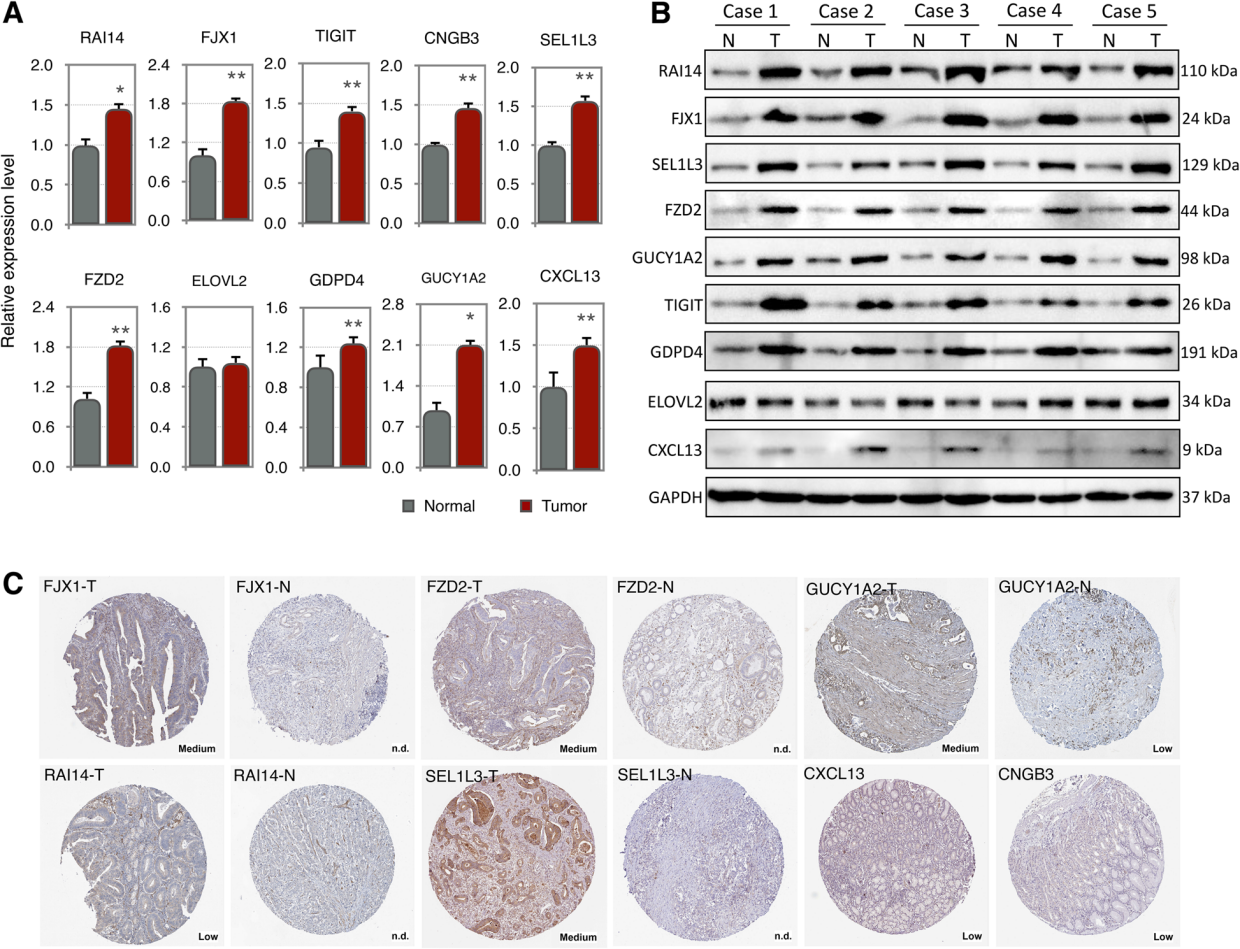
Expression validation of ten-mRNA prognostic features based on LNM-DEGs. **A** *q*RT-PCR validation of 10-mRNA expression levels in gastric cancer cell lines (AGS and MGC-803) and normal cell lines (GES-1). **B** Immunohistochemical map of protein expression in STAD and normal gastric tissues. Data were obtained from the Human Protein Atlas online database. **P < 0.01 and *P < 0.05

Ultimately, we verified the expression levels of the 10-mRNA signature using gastric cancer tissue and cell lines. As shown in Fig. 7A, the mRNA levels of *RAI14*, *GUCY1A2*, *CNGB3*, *FJX1*, *FZD2*, *ELOVL2*, and *GDPD4* were all expressed upregulated in gastric cancer tissues, except for *CXCL13*, whose expression level was not significantly different between gastric cancer tissues and normal cell lines. Similar results were observed in Western blot (Fig. 7B), except for CNGB3, which had no protein expression. We also detected the relative expression levels of the 10 mRNA signatures in gastric cancer cell lines, and the same results were found (Fig. S2). Furthermore, we obtained immunohistochemical results for 10 mRNA signatures in the HPA database to support their role in tumor tissues (Fig. 7C). The specific biological pathways involved in the 10-mRNA signature need to be verified in future in-depth studies.

## Discussion

STAD is the most common malignancy of the digestive system and has one of the highest incidence and mortality rates of all cancers worldwide. The effectiveness and strategy of cancer treatment often depend on the stage of cancer diagnosis. However, the development of STAD is a complex multistage process involving many genetic and epigenetic changes. Patients with STAD are mostly diagnosed at advanced stages, which leads to difficult treatment, poor prognosis, and high mortality. Molecular markers based on coding or non-coding genes have great potential in predicting cancer prognosis. The development of molecular markers that can effectively identify STAD at the LNM stage with a good prognosis is crucial for the treatment strategy and outcome of STAD.

The sensitivity or specificity of a single tumor marker used clinically is low and cannot reach the ideal state. Therefore, it is reasonable to realize the combined detection of multiple tumor markers and continuously monitor the change of tumor marker concentration to improve the sensitivity and specificity of screening and diagnosis, avoiding the waste of resources, and finally, achieving the purpose of improving the clinical treatment effect, prolonging the survival time of patients, and reducing the mortality of tumor patients. The change or not of tumor marker concentration can also monitor the clinical treatment effect and predict the chance of recovery. Tumor marker concentration will change significantly after effective treatment (such as complete tumor resection or chemotherapy). If it does not decrease as expected after surgery, it indicates poor treatment outcomes. In addition to this, changes in tumor marker concentrations can also predict or observe recurrence as part of a patient’s follow-up plan.

In this study, gene expression data of STAD samples and clinical information of patients were obtained from TCGA and GEO public databases. A total of 269 LNM-specific DEGs were identified by the bioinformatic mining method. Enrichment analysis revealed that these LNM-specific DEGs were mainly involved in cytokine-cytokine receptor interactions, neuroactive ligand-receptor interactions, and calcium signaling pathways. Multivariate Cox proportional hazard regression analysis of LNM-specific DEGs by a bivariate stepwise method was performed to screen 10 DEGs, including RAI14, GUCY1A2, CNGB3, FJX1, GDPD4, FZD2, ELOVL2, CXCL13, TIGIT, and SEL1L3, and the STAD prognostic signature was established based on them. Survival analysis and time-dependent ROC curve analysis of both thee TCGA-STAD test dataset and GEO validation dataset GSE84437 showed that the signature had good predictive value and predictive effect. Correlation analysis of the 10-mRNA signature with the clinical characteristics of STAD patients revealed that the signature was associated with the survival status of STAD patients in the LNM stage, advanced stage, late tissue grading, and advanced primary tumor stage. In addition, the 10-gene signature had a more effective and sensitive predictive effect compared with published biomarkers and can be used as an independent prognostic factor for STAD patients.

According to reported studies, the 10-mRNA signature gene was found to be associated with tumor progression and prognosis. silencing of RAI14 inhibits the proliferation and invasion of BC cells and the progression of esophageal cancer (EC) through the STAT3 pathway [23, 24]. High expression of GUCY1A2 in GC tissues and its association with poor patient prognosis could be used as a potential prognostic marker [25]. FJX1 can be a candidate diagnostic and prognostic biomarker for CRC patients; downregulation of FJX1 expression or neutralization of secretory FJX1 inhibited CRC cell proliferation and migration in vitro and is strongly associated with liver metastasis [26, 27]. Fzd2 played a role in BC cell mesenchymal-like stemness; targeting Fzd2 inhibits tumor cell recurrence, metastasis, and chemoresistance [28]. ELOVL2 inhibited cell proliferation, migration, and invasion in prostate cancer (PC) and may act as a novel tumor suppressor to attenuate tamoxifen resistance in BC; however, in renal cell carcinoma (RCC), ELOVL2 promoted cancer progression by inhibiting apoptosis [29–31]. CXCL13 promoted the formation of an immune microenvironment and enhanced the effects of PD-1 checkpoint blockade in plasmacytic advanced ovarian cancer (OC) [32]. TIGIT was an inhibitory receptor expressed by lymphocytes that played an important role in limiting the antitumor response process and the cancer-immune cycle [33]. SEL1L3, together with other key genes, has been used as a polygenic prognostic signature in patients with glioblastoma (GBM) or LUAD [34, 35]. The above reports imply a potential impact of the constructed 10-mRNA prognostic signature on STAD.

In this study, we constructed a 10-gene STAD prognostic signature based on LNM-specific DEGs, which have a good predictive effect on STAD prognosis at the LNM stage and have not been reported yet. This signature could be used as a potential biomarker to predict STAD prognosis and provide theoretical support for the mining of relevant therapeutic target drugs. The shortcoming of our current research was that more clinical data samples need to be collected to verify the validity and reliability of the prognostic signature because the STAD dataset involved in the study is from public databases. Meanwhile, the biological functions of prognostic signature genes in gastric cancer development also deserve further experimental studies to verify.

## Conclusion

Through a series of bioinformatics approaches to analyze the gene expression differences between STAD and normal tissues in the TCGA dataset, we obtained 269 LNM-specific DEGs. Enrichment analysis gave us insight into the biological functions and pathways that LNM-specific DEGs may be involved in. Subsequently, using the TCGA-STAD and GSE84437 datasets, we constructed a 10-mRNA prognostic signature based on LNM STAD-specific DEGs, including RAI14, GUCY1A2, CNGB3, FJX1, GDPD4, FZD2, ELOVL2, CXCL13, TIGIT, and SEL1L3. This prognostic feature has good sensitivity and accuracy in predicting the prognosis of STAD patients. Based on the current study, we will continue to explore prognostic genes and their potential biological functions.

## Supplementary Information

**Additional file 1: Fig. S1.** The LNM-DEGs associated with OS for STAD patients in TCGA Datasets based on uniCox (A) and multiCox (B) proportional hazard.

**Additional file 2: Fig. S2.** *q*RT-PCR validation of 10-mRNA expression levels in gastric cancer cell lines (AGS and MGC-803) and normal cell lines (GES-1). ** indicates *P* < 0.01, and * indicates *P* < 0.05.

**Additional file 3: Fig. S3.** The original images of WB in this study to detect the protein expression level of 10-mRNA signature and GAPDH which as the internal control.

**Additional file 4: Table S1.** Clinicopathological characteristics of training and test sets for STAD patients in datasets.

**Additional file 5: Table S2.** The sequence of primers in this study.

**Additional file 6: Table S3.** Identification of LNM-specific DEGs.

**Additional file 7: Table S4.** GO analysis of the LNM-specific DEGs.

**Additional file 8: Table S5.** GO analysis of the nonLNM-specific DEGs.

**Additional file 9: Table S6.** GO analysis of the coDEGs.

**Additional file 10: Table S7.** Pathway enrichment analysis of the LNM-specific DEGs.

**Additional file 11: Table S8.** Pathway enrichment analysis of the nonLNM-specific DEGs.

**Additional file 12: Table S9.** Pathway enrichment analysis of the coDEGs.

**Additional file 13: Table S10.** GSEA analysis of GO enrichment of DEGs related to the 10-mRNA signature.

**Additional file 14: Table S11.** GSEA analysis of KEGG pathways enrichment of DEGs related to the 10-mRNA signature.

**Additional file 15: Table S12.** GSEA analysis of Reactome pathways enrichment of DEGs related to the 10-mRNA signature.

**Additional file 16: Table S13.** PPI network associated with the 10-mRNA signature.

**Additional file 17: Table S14.** Pathway enrichment analysis of the PPI network associated with the 10-mRNA signature.

## Data Availability

The TCGA and GEO belong to public databases. The patients involved in the database have obtained ethical approval. Users can download relevant data for free for research and publish relevant articles. Our study is based on open-source data, so there are no ethical issues and other conflicts of interest.

## Abbreviations

AJCC: American Joint Commission
AUC: The area under the curve
BC: Bladder cancer
BP: Biological processes
CC: Cellular components
C-index: Harrell consistency index
co-DEGs: Co-expression DEGs
CRC: Cervical cancer
DCA: Decision curve analysis
DEGs: Differentially expressed mRNAs
EC: Esophageal cancer
FC: Fold change
FDR: False discovery rate
GC: Gastric cancer
GBM: Glioblastoma
GEO: The Gene Expression Omnibus
GO: Gene Ontology
GSEA: Gene set enrichment analysis
HPA: Human Protein Atlas
KEGG: Kyoto Encyclopedia of Genes and Genomes
IRGs: Immune-related gene signatures
LNM: Lymph node metastasis
LUAD: Lung adenocarcinoma
MCODE: Molecular complex detection
MF: Molecular functions
mRNAs: Messenger RNAs
KM analysis: Kaplan-Meier analysis
OC: Ovarian cancer
OS: Overall survival
PC: Prostate cancer
PPI: Protein-protein interaction
RCC: Renal cell carcinoma
ROC: Receiver operating characteristic curve
RS: Risk score
STAD: Stomach adenocarcinoma
STRING: The Search Tool for the Retrieval of Interacting Genes
TCGA: The Cancer Genome Atlas
