# Supplementary figures and images for: Identification of critical prognosis signature associated with lymph node metastasis of stomach adenocarcinomas
Source: World J Surg Oncol. 2023 Feb 23;21:61. doi: 10.1186/s12957-023-02940-y (PMC9948474; doi:10.1186/s12957-023-02940-y)

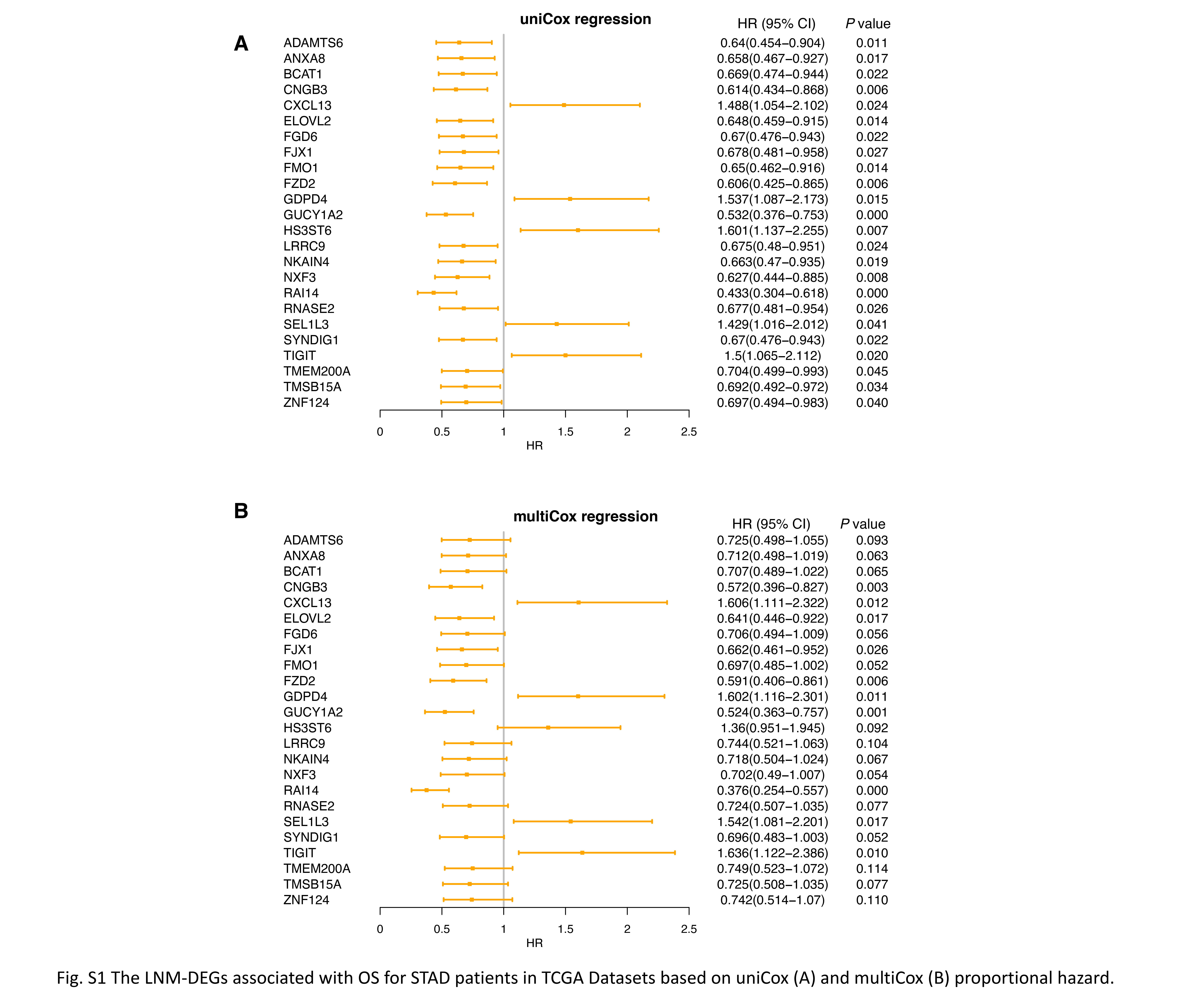

Supplement: Supplementary file 1 — Additional file 1: Fig. S1. The LNM-DEGs associated with OS for STAD patients in TCGA Datasets based on uniCox (A) and multiCox (B) proportional hazard. [file 12957_2023_2940_MOESM1_ESM.tif]

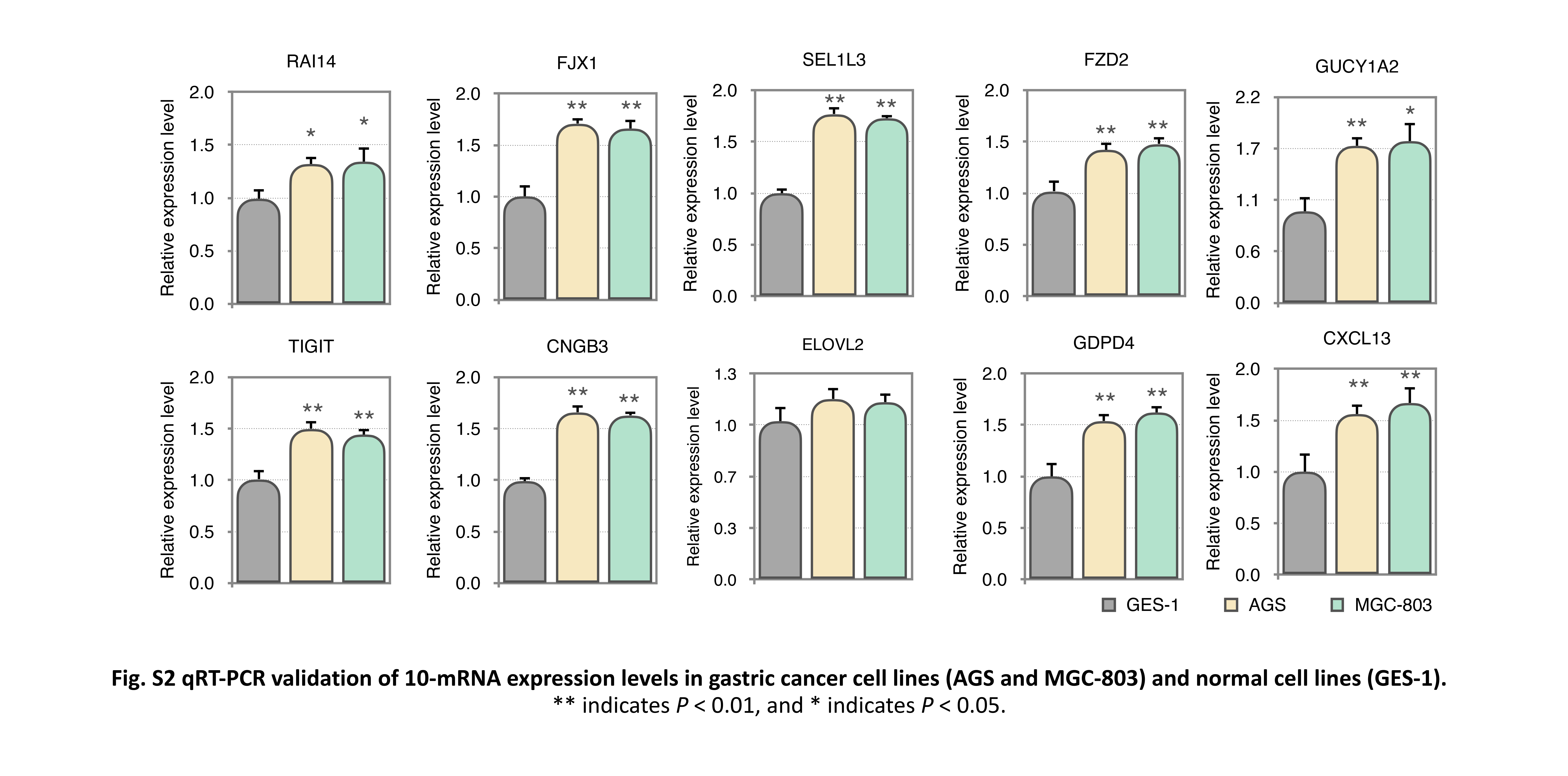

Supplement: Supplementary file 2 — Additional file 2: Fig. S2. qRT-PCR validation of 10-mRNA expression levels in gastric cancer cell lines (AGS and MGC-803) and normal cell lines (GES-1). ** indicates P < 0.01, and * indicates P < 0.05. [file 12957_2023_2940_MOESM2_ESM.tif]

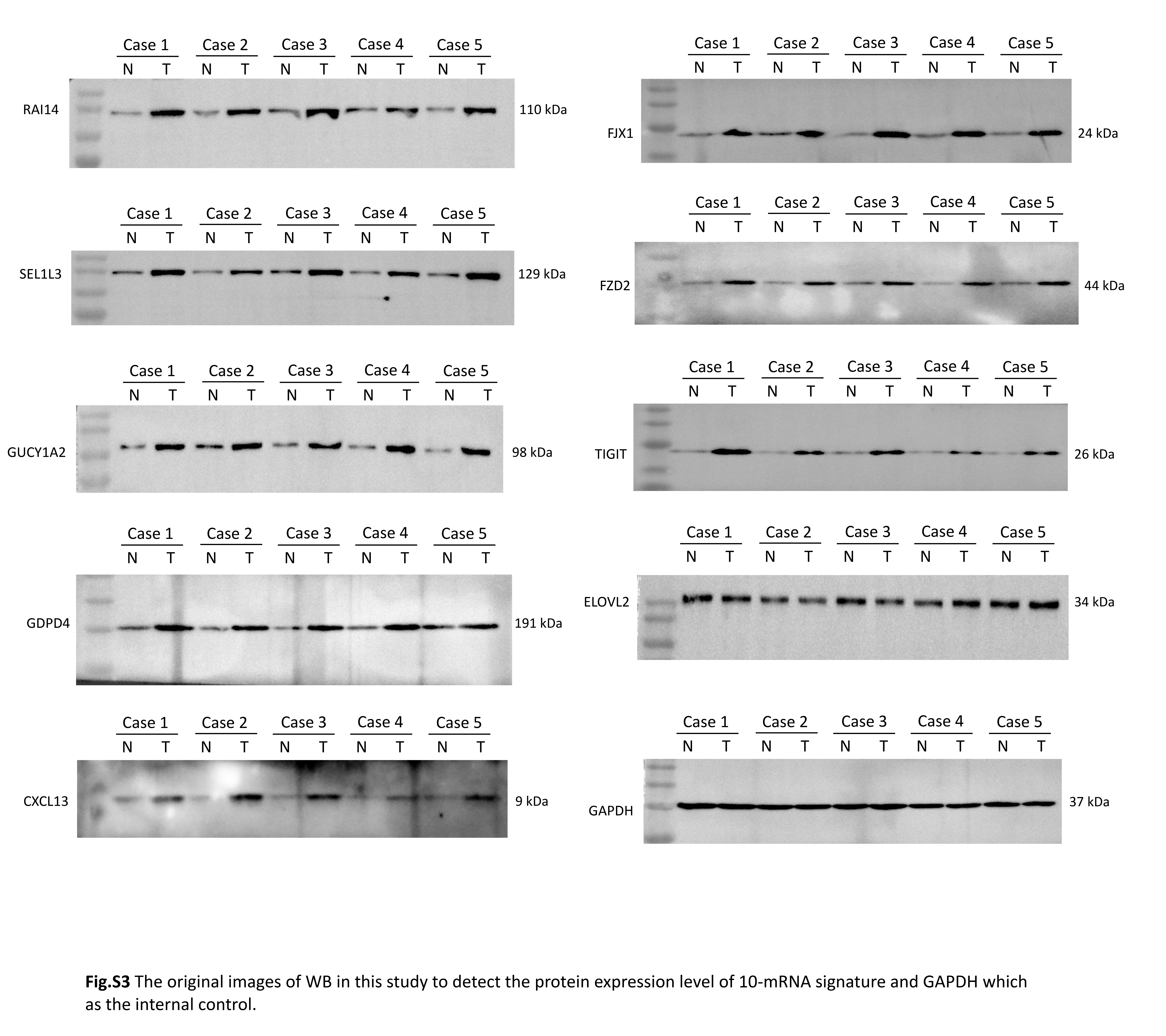

Supplement: Supplementary file 3 — Additional file 3: Fig. S3. The original images of WB in this study to detect the protein expression level of 10-mRNA signature and GAPDH which as the internal control. [file 12957_2023_2940_MOESM3_ESM.tif]
