# Supplementary material for: Identification of critical prognosis signature associated with lymph node metastasis of stomach adenocarcinomas
Source: World J Surg Oncol. 2023 Feb 23;21:61. doi: 10.1186/s12957-023-02940-y (PMC9948474; doi:10.1186/s12957-023-02940-y)
Supplement: Supplementary file 4 — Additional file 4: Table S1. Clinicopathological characteristics of training and test sets for STAD patients in datasets. [file 12957_2023_2940_MOESM4_ESM.docx]

**Table S1** Clinicopathological characteristics of training and test sets for STAD patients in datasets

| Clinical factors | | TCGA (%) | GSE84437 (%) |
| --- | --- | --- | --- |
| Stage | I | 46 (14.2) | n.d. |
|  | II | 104 (31.8) | n.d. |
|  | III | 143 (43.6) | n.d. |
|  | IV | 33 (10.3) | n.d. |
| T | T1 | 18 (5.5) | 11 (2.5) |
|  | T2 | 68 (20.9) | 38 (8.8) |
|  | T3 | 153 (47.0) | 92 (21.2) |
|  | T4 | 87 (26.7) | 292 (67.4) |
| N | N0 | 102 (32.4) | 80 (18.5) |
|  | N1 | 87 (26.4) | 188 (43.4) |
|  | N2 | 70 (21.2) | 132 (30.5) |
|  | N3 | 67 (20.0) | 33 (7.6) |
| M | M0 | 305 (93.6) | n.d. |
|  | M1 | 21 (6.4) | n.d. |
| Age | <=60 | 108 (33.2) | 194 (44.8) |
|  | >60 | 218 (66.8) | 239 (55.2) |
| Gender | Female | 125 (38.5) | 137 (31.6) |
|  | Male | 201 (61.5) | 296 (68.4) |
