# Supplementary material for: Identification of critical prognosis signature associated with lymph node metastasis of stomach adenocarcinomas
Source: World J Surg Oncol. 2023 Feb 23;21:61. doi: 10.1186/s12957-023-02940-y (PMC9948474; doi:10.1186/s12957-023-02940-y)
Supplement: Supplementary file 6 — Additional file 6: Table S3. Identification of LNM-specific DEGs. [file 12957_2023_2940_MOESM6_ESM.docx]

**Table S1 The sequence of primers in this study**

| Name of primers | sequence |
| --- | --- |
| RAI14 F | 5’- CTT GAA AGC GAA GTT CAG GAA GAG TGA CA -3’ |
| RAI14 R | 5’- AAG ATG GAA AGC GGT CTT GCC CTC A -3’ |
| FJX1 F | 5’- TAA GCA ACC TCT TCA GCC TGC AGT G -3’ |
| FJX1 R | 5’- TAC CCG GTA GCC GTG CAC CAA -3’ |
| SEL1L3 F | 5’- TTT AAC CTG GCC CTG CTA ATC GAG G -3’ |
| SEL1L3 R | 5’- TGG CTC CAG CAC CTT TCG TAC AGT T -3’ |
| GADPH F | 5’-CTT CGC TTC GCT ATC ATC GAC G-3’ |
| GADPH R | 5’-TCG AAC GTC TAG CGA AGC TA-3’ |
| GUCY1A2 F | 5’- TCG AAG GAA GAT TTC GTC CGA GTC CTT -3’ |
| GUCY1A2 R | 5’- TGA TGC TCT CGC CCA GCG AGT -3’ |
| TIGIT F | 5’- TGA TCT GGG CCC AGG GGC TGA -3’ |
| TIGIT R | 5’- TGA TAG AGC CAC CTT TCT CTG CAG AAA -3’ |
| CNGB3 F | 5’- GAA GCC TAT AGG AGA GAA CAA TGA GAA T -3’ |
| CNGB3 R | 5’- TCA CCT TTG TTT TCT TCC TGT GCT GT -3’ |
| GDPD4 F | 5’- CAG TGA ATA CTT TAA CTT TGA CTG GGT CAC T -3’ |
| GDPD4 R | 5’- CAA CAG CAA TGA AGA GTA GGC AGT CAG -3’ |
| ELOVL2 F | 5’- TGG ACA ATA TGT TTG GAC CGC GAG AT -3’ |
| ELOVL2 R | 5’- AGG TCT GTT CTT CAT ATA CTT GTT ACC CA -3’ |
| CXCL13 F | 5’- AAG TTC ATC TCG ACA TCT CTG CTT CTC AT -3’ |
| CXCL13 R | 5’- AGC TCT CTT GGA CAC ATC TAC ACC TCA A -3’ |
| FZD2 F | 5’- ATG ATC AAA TAC CTC ATG ACG CTC ATC -3’ |
| FZD2 R | 5’- TCA CAC GGT GGT CTC ACC GTG T -3’ |
